# β-Cyclodextrin-Immobilized Ni/Graphene Electrode for Electrochemical Enantiorecognition of Phenylalanine

**DOI:** 10.3390/ma13030777

**Published:** 2020-02-08

**Authors:** Feiyue Chen, Zhiqin Fan, Yangguang Zhu, Huifang Sun, Jinhong Yu, Nan Jiang, Shichao Zhao, Guosong Lai, Aimin Yu, Cheng-Te Lin, Chen Ye, Li Fu

**Affiliations:** 1 College of Science, Henan University of Technology, Zhengzhou 450001, China; chenfeiyue@nimte.ac.cn; 2 Key Laboratory of Marine Materials and Related Technologies, Zhejiang Key Laboratory of Marine Materials and Protective Technologies, Ningbo Institute of Materials Technology and Engineering (NIMTE), Chinese Academy of Sciences, Ningbo 315201, China; zhuyangguang@nimte.ac.cn (Y.Z.); sunhuifang@nimte.ac.cn (H.S.); yujinhong@nimte.ac.cn (J.Y.); jiangnan@nimte.ac.cn (N.J.); linzhengde@nimte.ac.cn (C.-T.L.); 3 Laboratory of Environmental Biotechnology, School of Environmental and Civil Engineering, Jiangnan University, Wuxi 214122, China; 4 Faculty of Materials Science and Engineering, Kunming University of Science and Technology, Kunming 650093, China; 5 Center of Materials Science and Optoelectronics Engineering, University of Chinese Academy of Sciences, Beijing 100049, China; 6 College of Materials and Environmental Engineering, Hangzhou Dianzi University, Hangzhou 310018, China; zhaoshichao@hdu.edu.cn; 7 Department of Chemistry, Hubei Normal University, Huangshi 435002, China; gslai@hbnu.edu.cn (G.L.); aiminyu@swin.edu.au (A.Y.); 8 Department of Chemistry and Biotechnology, Faculty of Science, Engineering and Technology, Swinburne University of Technology, Hawthorn VIC 3122, Australia

**Keywords:** graphene, plasma-enhanced chemical vapor deposition, β-cyclodextrin, enantiorecognition, phenylalanine

## Abstract

In this work, a Ni/graphene (Ni/G) electrode was designed and fabricated by plasma-enhanced chemical vapor deposition (PECVD) for the ultrasensitive recognition of d- and l-phenylalanine. Through a single-step PECVD process, the Ni/G electrode can achieve better hydrophilicity and larger catalytic surface area, which is beneficial for the electrochemical recognition of bio-objects. After surface modification with β-cyclodextrin, the Ni/G electrode can distinguish d-phenylalanine from l-phenylalanine according to a 0.09 V peak shift in differential pulse voltammetry tests. Moreover, this Ni/G electrode achieved a detection limit as low as 1 nM and a wide linear range from 1 nM to 10 mM toward l-phenylalanine, with great storage stability and working stability.

## 1. Introduction

Chiral compounds exhibit different pharmacokinetic and pharmacodynamic properties. For example, many pregnant women take the tranquilizer thalidomide and have fetal aberrations [1,2]. Thalidomide has been revoked at 1961 from the market. Later, it was found that the fetus teratogenicity was caused by its S-isomer. Therefore, the recognition of chiral compounds is very important in the biological and pharmaceutical fields. So far, high-performance liquid chromatography (HPLC) is the main technique for separating and quantifying enantiomers [3,4,5]. In addition, surface-enhanced Raman scattering spectroscopy [6,7], resonance Rayleigh scattering [8], and field-effect transistor [9] were used for identification as well. Although these methods are routinely used for accurate determination, they still suffer from some drawbacks such as long response time, high instrument cost, and sophisticated operation procedure. On the other hand, the electrochemical approach can be considered as an alternative method for enantiorecognition due to its high sensitivity, fast response, and low cost [9,10,11,12]. 

Common electrochemical-based methods for enantiorecognition can be divided into two strategies include molecularly imprinted polymers (MIP)-based biosensor and stereoselective material immobilized biosensor. MIP could be prepared as target-specific chiral stationary phases by binding target chiral compounds. However, the MIP preparation process involves time-consuming embossing and etching procedures. Moreover, the strong covalent bond between the polymer and the analyte creates a low efficiency of combination and dissociation in the process of molecular recognition and regeneration. It takes a long time to achieve the thermodynamic equilibrium and is not suitable for rapid discriminating [13]. In contrast, the stereoselective material immobilized biosensor can be directly used for discriminating sensing. However, the stereoselective materials such as cyclodextrin and chitosan have poor conductivity, which limited their enantiorecognition in the electrochemical field [10,12]. To solve this problem, conductive substrate materials were commonly used for forming complexes and were subsequently used for electrochemical enantiorecognition application. Among many conductive materials, graphene and its derivatives have been widely employed in electrochemical biosensors, owing to their versatile properties including good electrical conductivity, large specific surface area, and tunable functionalization [14]. As for chiral molecule sensors, most works were focused on the preparation of reduced graphene oxide/β-cyclodextrin (β-CD) complex from graphene oxide precursor due to its ease of functionalization and good dispersity in aqueous solution [12,15,16,17]. However, based on our previous experience [15], this strategy comes at the expense of the high intrinsic conductivity of graphene due to the incomplete reduction process and high surface adsorption of β-CD. Therefore, the combination of high-quality graphene with β-CD immobilization should be the best choice for electrochemical enantiorecognition with high sensitivity.

In this work, we constructed a Ni/graphene electrode (Ni/G) by plasma-enhanced chemical vapor deposition (PECVD). Plasma is able to introduce defects to graphene, which is beneficial for the enhancement of hydrophilicity for further elelctrochemical recognition. β-CD was immobilized on Ni/G as a modifier, whose cavity enable guest molecules to enter and form inclusion complexes, which is capable of discriminating between chiral amino acids according to the host–guest interaction deviation [18,19]. l-phenylalanine (Phe) and d-Phe were chosen to serve as chiral molecules. l-Phe is an essential amino acid for humans and need to be acquired from food. The lack or excess of l-Phe could result in a series of health problems such as depression, decreased alertness, memory problems, etc. In contrast, d-Phe has been proved its medicinal function as a chronic pain reliever [20]. The host–guest interaction between β-CD and enantiomers showed a clear peak separation for recognition. Due to the optimized performance of the Ni/G and surface modification of β-CD, the proposed β-CD/Ni/G electrode showed excellent enantiorecognition performance. 

## 2. Materials and Methods 

### 2.1. Preparation of β-Cyclodextrin/Ni/G Electrode

The preparation of the Ni/G electrode was based on the PECVD process in a tube furnace (BTF-1200C-Ⅱ-SL, AnHui BEQ Equipment Tech.) [21]. Briefly, a 1-mm-thick quartz slide has been used as an electrode substrate. First, 50-nm-thick nickel films were deposited on the quartz surface by an electron-beam evaporation machine (ULVAC Technologies Inc., Chigasaki-shi, Japan). After placing the Ni-coated quartz into the furnace, the furnace was heated to a 900 °C, and 10 sccm H_2_ was introduced as the protective gas. At the desired temperature, a gas mixture of H_2_/CH_4_ (10/20 sccm) at 0.36 Torr was introduced into the furnace for graphene growth, with the assistance of plasma at different power (50 W/100 W/150 W/200 W). After 10 min of growth, the Ni/G electrodes were rapidly cooled down to room temperature at a protective atmosphere (H_2_/Ar: 10/150 sccm). Then, an Ni/G electrode was immobilized on a plastic substrate and connected by silver paints to copper wire. The surface adsorption of β-CD was simply conducted by the immersion of Ni/graphene electrodes into 0.1 M β-CD solution for 6 min and dried by N_2_ gas. The resulting electrode was denoted as β-CD/Ni/G.

### 2.2. Chemicals and Characterizations

All reagents used were analytical grade and used as received. The quality of the graphene layer on the Ni/G electrode surface was characterized by Raman spectroscopy (Renishaw plc, Wotton-under-Edge, Gloucestershire, UK, laser wavelength: 532 nm). The morphology of the Ni/G electrode was characterized by a field-emission scanning electron microscopy (FE-SEM, QUANTA FEG 250, FEI, Hillsboro, OR, USA) and an atomic force microscope (AFM). All electrochemical measurements were conducted using a CHI 760E electrochemical workstation with a conventional three-electrode system comprised of platinum wire as the auxiliary electrode, a 3 M Ag/AgCl electrode as the reference, and prepared β-CD/Ni/G as the working electrode.

### 2.3. Electrochemical Enantiorecognition of Phenylalanine Enantiomers

For electrochemical enantiorecognition, the prepared β-CD/Ni/G/ was immersed into a 0.1 M phosphate buffer solution (PBS) containing 0.1 mM l-Phe or d-Phe for 2 min. Then, differential pulse voltammetry (DPV) was performed. The pulse width was 0.05 s, pulse period was 0.5 s, and amplitude was 50 mV. The electrochemical enantiorecognition of Phe enantiomers is based on the difference of oxidation peak potential of l-Phe and d-Phe.

## 3. Results and Discussions

**Figure 1:**
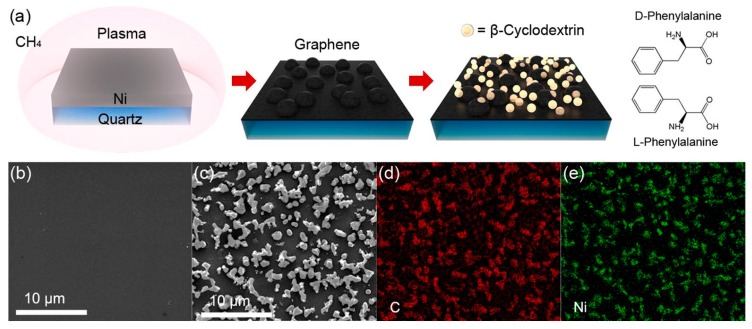
(**a**) Schematic diagram of β-cyclodextrin (β-CD)/Ni/G enantiorecognition biosensor fabrication. (**b**) SEM images of as-deposited Ni layer (**c**) SEM images of the Ni/G electrode. (**d**,**e**) The energy-dispersive spectroscopy (EDS) mappings of C and Ni of (**c**).

The schematic diagram of the electrochemical enantiorecognition biosensor fabrication was illustrated in Figure 1a. Briefly, the methane gas was decomposed during the PECVD, and graphene film was synthesized on Ni-coated quartz, followed by a necessary wiring and insulation process to get the Ni/G electrode. After that, β-CD molecules were modified on Ni/G electrode through van der Walls interaction by immersing into β-CD solution. Then, l-Phe or d-Phe molecules were trapped on the β-CD/Ni/G surface by the host–guest interactions via dipping the biosensor into an analyte solution. Owing to different steric hindrances of the l-Phe trapped β-CD and d-Phe trapped β-CD, the biosensor is expected to perform different electrochemical behavior [22,23]. Meanwhile, the excellent conductivity of the graphene could amplify electrochemical signals for improving the sensing performance [24]. 

In the PECVD process, different plasma power ranging from 50 to 200 W was applied, and four kinds of Ni/G electrodes were obtained. The morphology of the Ni film and Ni/G is shown in Figure 1b,c by SEM characterization. A uniform surface of Ni film can be observed before the graphene growth. While after the PECVD process, an uneven surface with many micro-scale particles is exhibited on Ni/G electrodes. Note that Ni/G electrodes with different plasma power have similar morphology. Furthermore, energy-dispersive spectroscopy (EDS) analysis was executed in mapping scanning mode in the same region of Figure 1c, and the results of the C and Ni elements are shown in Figure 1d,e. Based on the EDS mapping, the element distribution is generally consistent with SEM image. 

**Figure 2:**
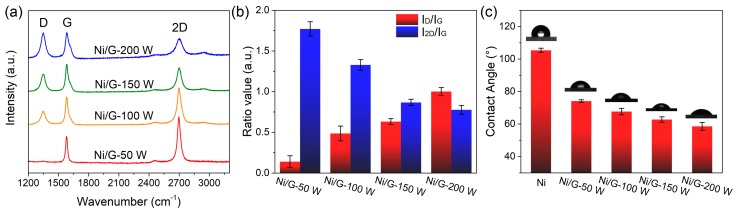
(**a**) Raman spectra of graphene of Ni/G electrode formed under different plasma power. (**b**) Id/I_G_ ratio and I_2D_/I_G_ ratio of Figure 2a. (**c**) Water contact angle of as-deposited Ni layer and Ni/G electrodes.

The quality of the formed graphene layer was characterized by Raman spectroscopy. Figure 2a shows the Raman spectra of graphene films grown on the top of the Ni layer with different plasma power. All spectra exhibit three characteristic peaks at 1350 cm^−1^ (D-band), 1581 cm^−1^ (G-band), and 2695 cm^−1^ (2D-band) [25,26,27,28]. D-band is attributed to the lattice disorder in graphene structrure. G-band is formed by the stretching vibration of sp^2^ carbon atoms, and the 2D-band is caused by second-order two phonons cattering. The narrow full width at half maxima (FWHM) of 2D-band (37.8–42.1 cm^−1^) indicates that the layer number of formed graphene is less than 5 [29]. The Raman spectrum of graphene of Ni/G-50 W is very close to single-layer graphene synthesized by conventional high-temperature CVD, due to its tiny D-band and intense 2D-band [30]. The peak intensity ratio of D-band to G-band (Id/I_G_) is corresponding to the defects degree of graphene, and the peak intensity ratio of 2D-band and G-band (I_2D_/I_G_) can reflect the thickness of graphene. The variations of the Id/I_G_ ratio and I_2D_/I_G_ ratio are calculated and summarized in Figure 2b. With the rise of plasma power from 50 to 200 W, the Id/I_G_ ratio of Ni/G electrodes gradually increased from 0.13 to 0.99, while the I_2D_/I_G_ ratio decreased from 1.74 to 0.77. Therefore, graphene synthesized with larger plasma power not only has more defects, but also more layer numbers. This can be attributed to two main functions of plasma during the PECVD process. One is to provide the activation energy for methane decomposition, so as to increase carbon atom concentration and accelerate graphene growth [21]. The other is to bombard the graphene surface to produce defects. As the graphene defects can lead to changes in hydrophilicity, contact angles of Ni film and Ni/G electrodes were measured via contact angle goniometer (Figure 2c). Deposited Ni film is hydrophobic with a contact angle of ~105°, while Ni/G electrodes are hydrophilic. With the further introduction of defects by higher power plasma, the hydrophilicity of Ni/G increased and contact angle gradually decreased from 72° to 58°.

**Figure 3:**
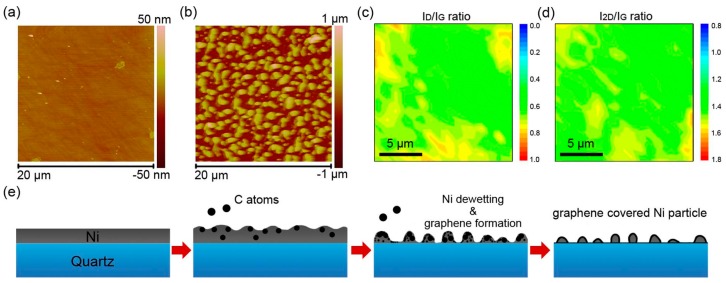
(**a**,**b**) Atomic force microscope (AFM) images of the surface morphology of as-deposited Ni layer and after Ni/G electrodes. (**c**,**d**) Raman mapping of Id/I_G_ ratio and I_2D_/I_G_ ratio of (**b**). (**e**) Scheme of change of surface morphology and graphene fabrication during the plasma-enhanced chemical vapor deposition (PECVD) process.

Atom force microscope (AFM) was used to analyze the 3D surface topography of Ni film and Ni/G electrode. As deposited through electron beam evaporation, Ni formed a homogeneous film with a low roughness of 0.76 nm (Figure 3a). After PECVD, Ni/G electrode exhibited a granular surface with a roughness of 123 nm (Figure 3b). As mentioned in Figure 1c, there was little difference of the surface morphology of the Ni/G electrode with the change of plasma power. Therefore, the variation of morphology was the Ni dewetting on quartz surface, which was caused by the high temperature of PECVD. To investigate the overall growth condition of graphene, the Raman in-plane Id/I_G_ and I_2D_/I_G_ mapping was collected (take Ni/G-100 W as an example). As shown in Figure 3c,d, the Id/I_G_ ratio is generally ranging from 0.6 to 0.8, and the I_2D_/I_G_ is ranging from 1.2 to 1.5. According to the result, graphene on Ni/G-100 W is few-layer defect-containing graphene. Furthermore, due to the smooth gradient of Raman mapping, graphene was integrally continuous even on such a granular surface. Therefore, the schematic diagram of graphene growth on 100-nm-thick Ni-deposited quartz through PECVD was concluded and shown in Figure 3e. Firstly, the introduced methane gas decomposited to carbon atoms with the coordination of plasma and high temperature as the energy supply. Then, C atoms dissolved into Ni and formed solid solution [31]. Meanwhile, the dewetting of Ni film occurred on quartz surface along with some evaporation of Ni atom. Afterwards, saturated C atoms precipitated out of Ni, nucleated, and grew into defect-containing graphene [21,32]. Finally, the Ni/G electrode was fabricated with a graphene-clad Ni particle structure.

**Figure 4:**
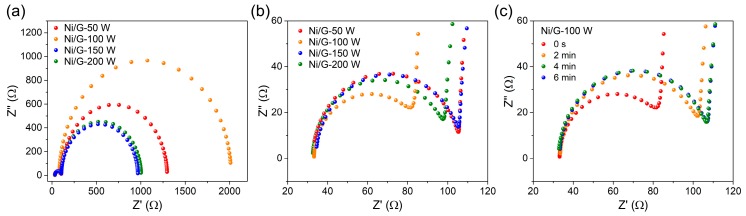
(**a**) Nyquist diagrams of Ni/G-50 W, Ni/G-100 W, Ni/G-150 W, and Ni/G-200 W in 5 mM of K_3_[Fe(CN)_6_] and 0.1 M KCl. (**b**) Enlarged view of high-frequency region of (**a**). (**c**) Nyquist diagrams of Ni/G-100W after different periods of β-CD adsorption.

The electrochemical behaviors of the electrodes were investigated by K_3_[Fe(CN)_6_] probes using electrochemical impedance spectroscopy (EIS). As shown in Figure 4a, the Nyquist plots of all electrodes exhibited small semicircle portions at high-frequency range, which corresponds to the electron transfer-limited process. The EIS data were fitted through an equivalent circuit model as R(CR)(CR), and the fitting results are shown in Table 1. The charge transfer resistance (R_t_) of Ni/G electrodes is arranged in such an order: Ni/G-50 W (74.1 Ω) ˃ Ni/G-150 W (72.9 Ω) ˃ Ni/G-200 W (68.1 Ω) ˃ Ni/G-100 W (55.6 Ω), which corresponds to their radius in enlarged view (Figure 4b). These results reflect competition between hydrophilicity and electrical properties due to the introduction of defects in graphene. On one hand, the defects of graphene could improve its hydrophilicity, which would enhance the interfacial affinity between electrode and electrolyte, benefiting to the electron transfer on the interface. On the other hand, the introduction of defects damaged the atom structure of graphene, which caused an overall loss of its intrinsic electrical properties. According to the results, Ni/G-100 W has been regarded as the optimized electrode for further enantiorecognition investigation. Surface β-CD modification is a crucial step for fabricating enantiorecognition sensor due to its selective chiral recognition ability [23]. In this work, β-CD functionalization was simply achieved by immersing Ni/G-100 W into a β-CD solution for 6 min. Figure 4c shows the Nyquist diagrams of Ni/G after different periods of β-CD adsorption. It can be seen that the charge transfer resistance of the Ni/G electrode increased as the adsorption increased from 0 min to 6 min and leveled off when a longer adsorption time was applied, suggesting that most of the β-CD molecules could be adsorbed on the Ni/G-100 W surface within 4 min.

**Figure 5:**
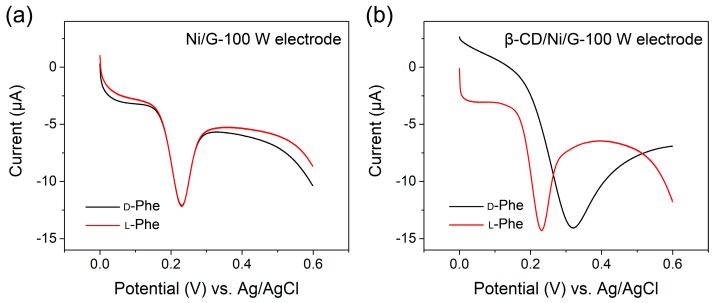
Differential pulse voltammetry (DPV) curves of 1 μM l-Phe and d-Phe at (**a**) Ni/G-100W and (**b**) β-CD/Ni/G-100W.

To verify the performance of the β-CD-modified Ni/G-100 W electrode, l-Phe and d-Phe recognition was investigated through a DPV test. As the control group, Ni/G-100 W electrode without β-CD was firstly tested. As shown in Figure 5a, the DPV curves of l-Phe and d-Phe of phenylalanine are in similar shape with one major oxidation peak, while their peaks almost coincide with each other at approximately 0.23 V versus Ag/AgCl. This result shows that l-Phe and d-Phe have similar apparent activation energy of their oxidation reaction at the surface of Ni/G-100 W without β-CD. Under same condition, the DPV curves of l-Phe and d-Phe measured on a β-CD-modified Ni/G-100W electrode are shown in Figure 5b. The oxidation peak position of L-Phe maintains at approximately 0.23 V versus Ag/AgCl, while that of d-Phe shifts to approximately 0.32 V versus Ag/AgCl. Moreover, the oxidation peak current of the l-Phe is more distinct than that of the d-Phe. the β-CD-modified Ni/G-100W electrode exhibited good enantiomeric resolution for l-Phe and d-Phe, which can be ascribed to the favorable H-bonds formation between the amino group of l-Phe and the secondary hydroxyl groups of β-CD [12,32,33]. Thus, l-Phe has smaller steric hindrances than d-Phe toward the Ni/G-100 W electrode, resulting in an increase of the apparent activation energy of oxidation reaction of d-Phe, which corresponded to its peak shift to higher potential. This electrode based on PECVD graphene exhibited an ultralow detection limit of 1 nM toward l-Phe, with the linear range from 1 nM to 10 mM (Figures S1 and S2). The detection limit is two orders lower than that of the electrode based on reduced graphene oxide [12], reflecting an excellent electrochemical activity. Some previous literatures indicated that reduced graphene oxide is more electrochemically active than pristine graphene because of the defective structure with abundant reactive sites [34]. However, CVD graphene has higher electrical conductivity and stronger binding toward a modifier and analyte [35]. Moreover, owing to the plasma bombardment during the PECVD process, our graphene combines the advantages of defective structure and CVD graphene, and it leads to better properties and improved sensing performance. Additionally, the sensing performance of Ni/G-100 W electrode was maintained over ≈95% after 7 days and the detection deviation is smaller than 7% during repeated experiments, exhibiting good storage stability and working stability (Figures S3 and S4). As a consequence, with the chiral selectivity given by β-CD, Ni/G-100 W electrode performed excellent sensing property for distinguishing l-Phe and d-Phe.

## 4. Conclusions

In this work, we proposed a Ni/graphene hybrid electrode by PECVD for the electrochemical enantiorecognition of phenylalanine. Plasma not only acted as a kind of power source to promote the methane decomposition, but also as a defect initiator to graphene. An appropriate amount of defects in graphene is conducive to better hydrophilicity for electrochemical electrode. Grown under optimized 100 W plasma, the Ni/G electrode has the lowest interfacial charge transfer resistance at 55.6 Ω. Modified with β-CD, this Ni/G electrode can distinguish d-Phe from l-Phe with a notable 0.09 V peak shift in DPV curves. In all, our research provides a possible method to fabricate an enantiorecognition sensor based on functionalized CVD graphene for higher sensitivity and specificity.

## Figures and Tables

**Table 1:** Fitting results of electrochemical impedance spectroscopy (EIS) spectra in Figure 4a.

Sample	R_s_ (Ω)	C_c_ (F)	R_c_ (Ω)	C_dl_ (F)	R_t_ (Ω)
Ni/G-50W	32.8	1.82 × 10^−4^	1188	2.77 × 10^−7^	74.1
Ni/G-100W	33.3	2.77 × 10^−4^	1928	4.88 × 10^−6^	55.6
Ni/G-150W	34.3	1.93 × 10^−4^	859.5	4.86 × 10^−7^	72.9
Ni/G-200W	32.7	2.34 × 10^−4^	902.2	1.22 × 10^−6^	68.1

## Supplementary Materials

The following are available online at https://www.mdpi.com/1996-1944/13/3/777/s1, Figure S1: DPV curves of different concentrations of l-Phe, Figure S2: The peak current as a function of added concentrations of l-Phe, Figure S3: The change of electrode performance during long-term storage, Figure S4: DPV curves of repeated experiments with an l-Phe concentration of 0.1 mM.
