# Supplementary material for: β-Cyclodextrin-Immobilized Ni/Graphene Electrode for Electrochemical Enantiorecognition of Phenylalanine
Source: Materials (Basel). 2020 Feb 8;13(3):777. doi: 10.3390/ma13030777 (PMC7040631; doi:10.3390/ma13030777)
Supplement: Supplementary file 1 [file materials-13-00777-s001.pdf]

materials

MDPI

Supplementary Materials

β-Cyclodextrin-Immobilized Ni/Graphene Electrode for Electrochemical Enantiorecognition of Phenylalanine

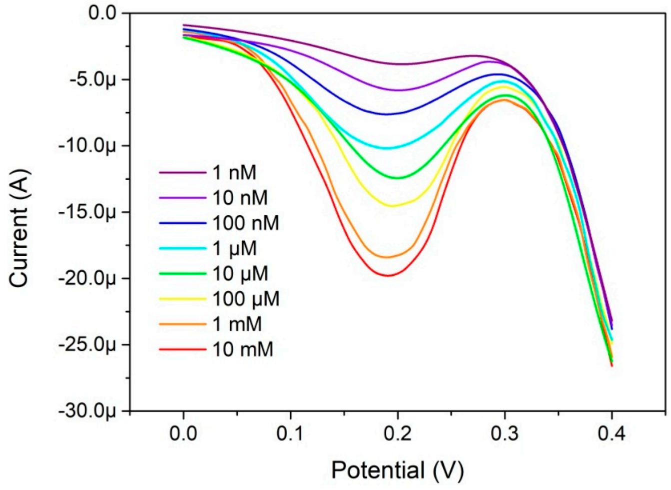

Figure S1. DPV curves of different concentrations of L-phenylalanine.

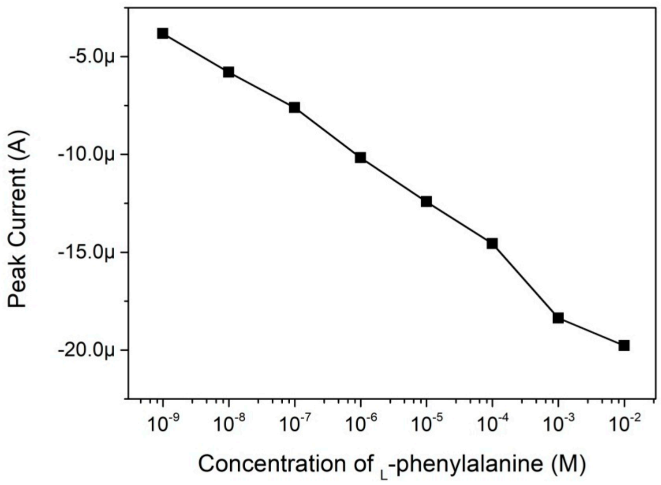

Figure S2. The peak current as a function of added concentrations of L-phenylalanine.

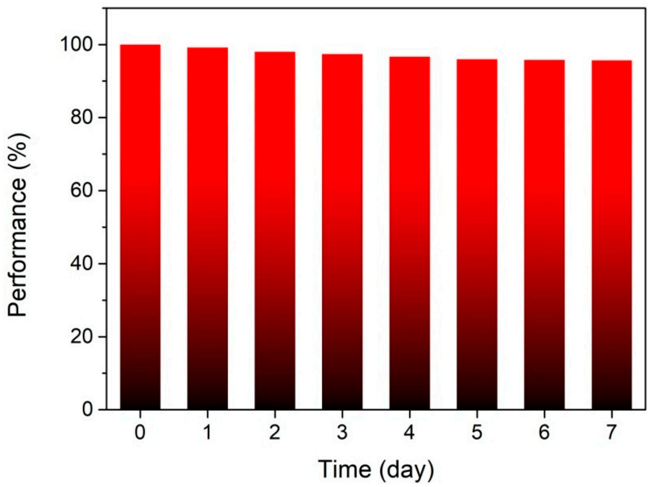

Figure S3. The change of electrode performance during long-term storage.

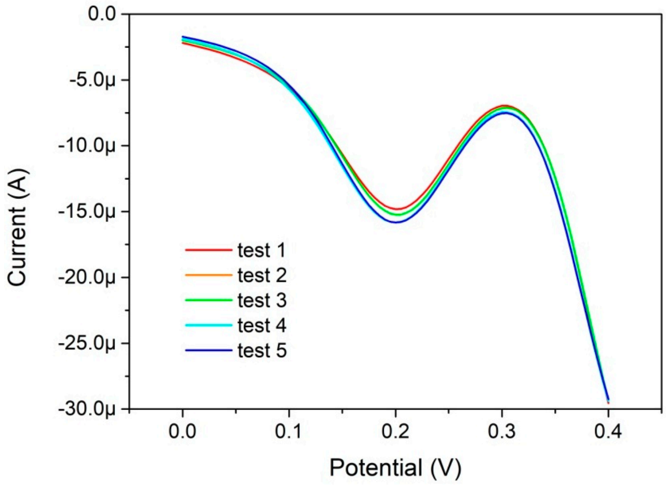

Figure S4. DPV curves of repeated experiments with an L-phenylalanine concentration of 0.1 mM.

© 2020 by the authors. Submitted for possible open access publication under the terms and conditions of the Creative Commons Attribution (CC BY) license (http://creativecommons.org/licenses/by/4.0/).
